# Barriers to Insecticide-Treated Mosquito Net Possession 2 Years after a Mass Free Distribution Campaign in Luangwa District, Zambia

**DOI:** 10.1371/journal.pone.0013129

**Published:** 2010-11-10

**Authors:** David A. Larsen, Joseph Keating, John Miller, Adam Bennett, Cynthia Changufu, Cecilia Katebe, Thomas P. Eisele

**Affiliations:** 1 Department of International Health and Development, Tulane University School of Public Health and Tropical Medicine, New Orleans, Louisiana, United States of America; 2 Program for Appropriate Technology in Health (PATH) Malaria Control and Evaluation Partnership in Africa (MACEPA), Lusaka, Zambia; 3 Society for Family Health, Lusaka, Zambia; 4 National Malaria Control, Ministry of Health, Lusaka, Zambia; Kenya Medical Research Institute, Kenya

## Abstract

**Background and Methods:**

Roll Back Malaria set the goal of 100% of households in malaria endemic countries in Africa owning an insecticide-treated mosquito net (ITN) by 2010. Zambia has used mass free distribution campaigns and distribution through antenatal care (ANC) clinics to achieve high coverage.

**Methodology and Principal Findings:**

We conducted a probability survey of 801 households in 2008 to assess factors associated with households that lacked an ITN after mass distribution. Community perceptions of barriers to ITN access were also obtained from in-depth interviews with household heads that reported not owning an ITN. Nearly 74% of households in Luangwa district reported owning ≥1 ITN. Logistic regression showed households without a child <5 years old during the ITN distribution campaigns were twice as likely to not have an ITN as those with a child <5 during distribution (Adjusted odds ratio (AOR)  = 2.43; 95% confidence interval (CI): 1.67–3.55). Households without a woman who attended an ANC in the past 2 years were more likely to be without ITNs compared to households with a woman who attended an ANC in the past 2 years (AOR  = 1.52; 95% CI: 1.04–2.21). In-depth interviews with heads of households without an ITN revealed that old age was a perceived barrier to receiving an ITN during distribution, and that ITNs wore out before they could be replaced.

**Conclusions and Significance:**

Delivery of a large number of ITNs does not translate directly into 100% household coverage. Due to their design, current ITN distribution strategies may miss households occupied by the elderly and those without children or ANC access. ITN distribution strategies targeting the elderly, those with limited access to distribution points, and others most likely to be missed are necessary if 100% ITN coverage of households is to be achieved.

## Introduction

Insecticide-treated mosquito nets (ITNs) are an effective strategy for preventing malaria in children and pregnant women [1], [2]. With increased funding from international donors [3], [4], [5], there has been a call for “scale-up for impact” in malaria endemic countries of Africa with a focus on achieving high coverage of effective interventions [5]. Within this framework the goal is to achieve universal coverage of ITNs in malaria endemic settings, defined as 100% of households with ≥1 ITNs, with 80% use by the end of 2010 [5], [6].

Countries have relied on a number of different ITN delivery strategies to scale-up ITN coverage, including mass free distribution, routine free or subsidized distribution through channels such as antenatal care (ANC) clinics, or the sale of ITNs through private retailers at a subsidized price [7], [8]. Most countries have relied on a combination of such approaches to meet national and global targets [9]. Regardless of the distribution strategy, achieving 100% coverage of households possessing ≥1 ITN will be challenging, especially in rural areas of Africa where the burden of malaria is often greatest and access to health delivery mechanisms are limited. Inevitably, certain segments of the population are missed during distribution efforts due to multiple factors, including children or pregnant women absent from the house, the inability to purchase an ITN, a lack of access to health care delivery points, and limited malaria-related knowledge, attitudes and practices [10], [11], [12], [13], [14], [15].

Zambia was an early recipient of support from donors to scale-up malaria control efforts across the country, which included funding since 2005 from the Global Fund to Fight AIDS, TB and Malaria, the Presidents Malaria Initiative (PMI), the World Bank Booster Program and the Bill and Malinda Gates Foundation [16]. The Zambia Ministry of Health adopted the scale-up for impact approach to rapidly achieve 80% coverage of ITN use by vulnerable populations by 2008, and has subsequently pushed for universal coverage to meet end-of-decade targets [17]. While children <5 years old and pregnant women were prioritized by initial ITN distributions, distribution has shifted toward mass distribution with the aim of achieving wide-scale coverage of all age and population groups. Mass distributions targeting all households are currently supplemented by routine distribution through ANC clinics in Zambia, which ensures high coverage of households with children <5 years old and pregnant women. Luangwa was one of the first districts in Zambia to be targeted for the rapid scale-up of ITNs.

This paper examines the factors related to households that were missed by the ITN distribution campaigns in rural Luangwa District, Zambia 2005–2008. Quantitative findings are supplemented with qualitative data that explore issues related to why particular households were missed.

## Methods

### Study Site and Net Campaign

The study was conducted in Luangwa district Zambia in 2008–2009, a remote area lying at the convergence of the Luangwa and Zambezi rivers. Approximately 34,000 people live in Luangwa district. The entire district is considered rural according to the Central Statistics Office of Zambia; a single municipality is located at the southernmost point of the district (Luangwa Boma). The population is served by 9 rural health centers, 2 of which have both inpatient and outpatient services. Fishing is the principal economic activity, and is supplemented by agriculture and animal husbandry.

Malaria transmission is endemic, with peaks in transmission typically occurring from April to June. Malaria parasite infection prevalence in children <5 years old was 7% at the end of the peak transmission period in 2008; infection prevalence ranged from 0.6% near Luangwa Boma to 18.2% in the northern part of the district [18].

A total of 2,100 ITNs and 7,000 long-lasting ITNs (LLINs) were distributed free of charge in November 2005 and February 2006, respectively, as part of a national campaign to provide 1 net to every household in Luangwa District. The campaigns were carried out by the Luangwa District Health Management Team and the National Malaria Control Centre, with support from the Malaria Control and Evaluation Partnership in Africa (MACEPA) project and many other partners. Before the distribution, community health workers and malaria agent volunteers registered each household. Representatives of each household were asked to travel to one of the 9 rural health centers or their associated community health worker (CHW) health posts to receive the free ITNs. In the fall of 2006, an additional 7,000 LLINs were made available to the district health office for resell at ANC clinics at a subsidized price of 3,000 Kwacha (equivalent to $0.50–$0.70 US). In total, over 16,000 ITNs and LLINs were distributed from 2005–2006, sufficient to achieve a ratio of 3 nets per household in the district.

### Data Collection

We conducted a district-representative household survey at the end of the peak malaria transmission season in April-May 2008, as described elsewhere [18]. In summary, 801 households were selected through simple random sampling of a complete digitized listing of all 3,998 households in the district for data collection. The survey followed the established Zambia Malaria Indicator Survey (MIS) protocol for collecting data on household characteristics, women of reproductive age and their children. Data on ITN household possession and use were ascertained from a net roster, while information on malaria-related knowledge, beliefs and practices were obtained from a standardized women's questionnaire [19].

Individual in-depth interviews were conducted in February 2009 with 10 heads of households out of a possible 212 households that reported not owning an ITN during the household survey to explore reasons for not owning ≥1 ITN. Their respective community health workers were also interviewed to obtain a perspective from a trusted member of the community regarding the ITN distribution in that community. In-depth interviews were conducted in Nyanja, the local language spoken in Luangwa; interviews were recorded and then transcribed into English. Free ITNs were given to the heads of households who participated in the interviews once finished.

### Data Analysis

Mosquito nets were classified as ITNs using standard definitions if they were one of the following: LLINs, ITNs purchased in the previous year, or mosquito nets that had been treated with insecticide in the previous year. Households that reported or were observed to have no ITNs were categorized as not owning ITNs. We calculated the number of ITNs in circulation in the district by multiplying the mean number of ITNs per household by the total number of households in the district. Chi-square statistics and student t-tests were used to investigate differences in ITN ownership among sub-groups of the household population. We used a random effects logistic regression model to identify factors related to lack of ITN ownership. The following factors were investigated in relation to lack of ITN ownership: the presence of a child who would have been <5 during the mass campaign (estimated from the household roster); the presence of a woman who attended ANC in the past 2 years in the household (ascertained from the women's questionnaire); socioeconomic status as measured by wealth quintiles derived from an asset index based on principle component analysis including such assets as type of drinking water, type of flooring, ownership of a bicycle or motorcycle, and presence of electricity in the home [20]; and the distance to the nearest health facility in kilometers. The presence of a child who would have been <5 at the time of the distribution campaign and the presence of a woman in the household who attended ANC reflect a measure of access to ITNs as they were the target of the 2005–2006 distribution campaigns in Luangwa. Distance to the nearest health facility captured access to the distribution points, while also acting as a proxy for access to information on ITNs. Receipt of a free ITN by household was dependent upon availability of ITNs at the health center; to account for correlated observations, we included a categorical variable for health center as a random intercept in the model. The same model with household ownership of any type of mosquito net, classified as an ITN or not, as the dependent variable was used to see if results differed due to information bias in the classification of ITNs. All results were considered statistically significant at the 5% level.

We transcribed the voice-recorded in-depth interviews and manually coded responses into themes relating to ITN ownership using standard anthropological methods [21]. Common themes related to possession of ITNs were interpreted and grouped together accordingly. The interviews from heads of households were interpreted separately from the interviews with community health workers.

Stata® version 11 (Stata Corporation, College Station, Texas) was used to analyze the household survey data. ArcGIS® was used to calculate distance of households to health facilities.

### Ethics Statement

Ethical approval for this study protocol was obtained through the Institutional Review Boards (IRB) of Tulane University, the University of Zambia, and the Program for Appropriate Technology in Health (PATH). Participants in the household survey gave informed written consent. Participants for the in-depth interviews gave informed recorded verbal consent.

## Results

**Figure 1:**
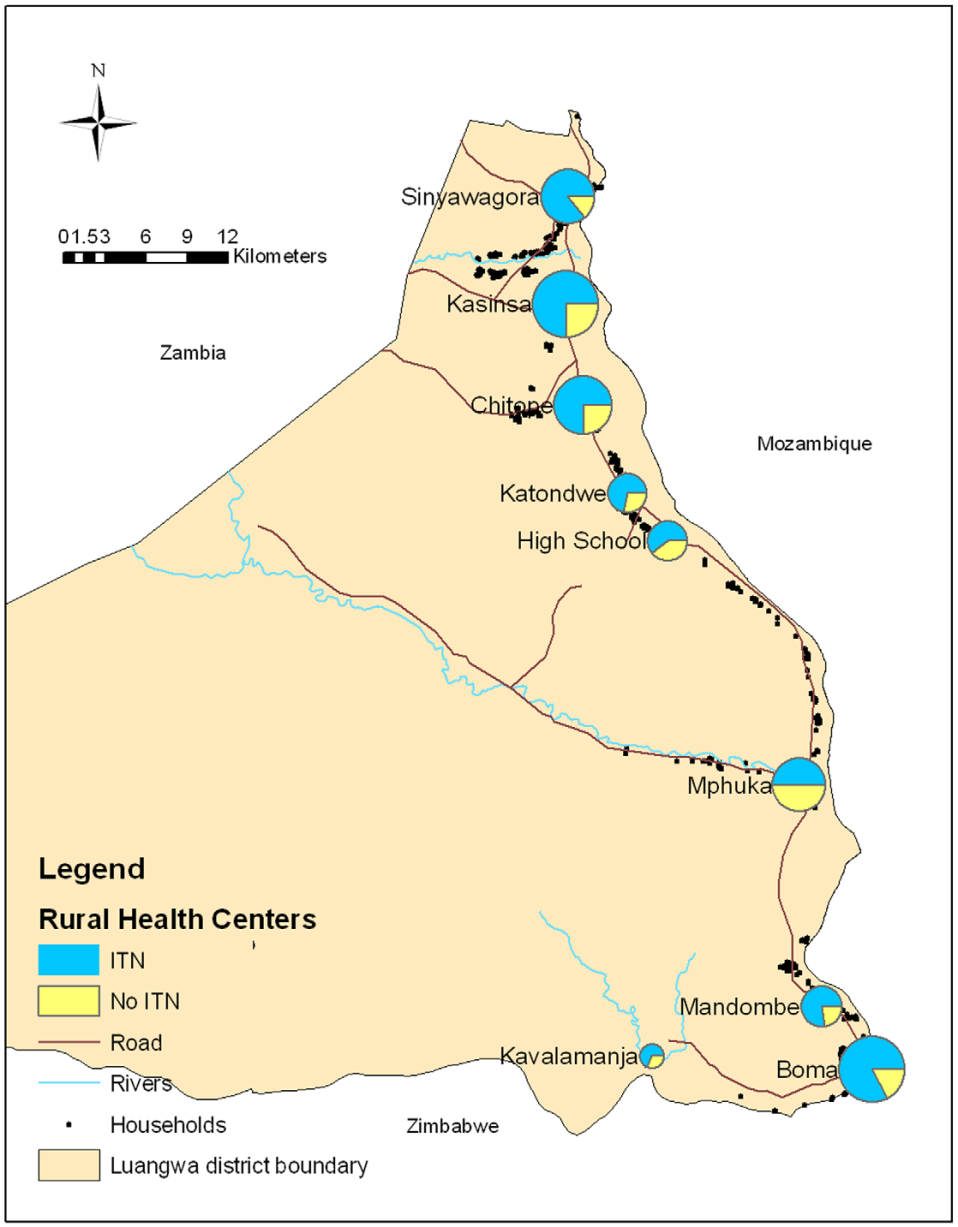
Ownership of ITNs by rural health center catchment areas, Luangwa District Zambia 2008. Pie size represents relative population size of catchment area.

Although 16,000 ITNs were reportedly delivered to Luangwa district from 2005–2006, 6,118 ITNs (95% CI  = 5,749–6,487) were estimated to be present in households from the 2008 survey. Overall, 73% of all households reported owning ≥1 ITN; 82% of households with a child <5 reported owning ≥1 ITN. Ownership of an ITN varied by health center catchment area, with ITN ownership ranging from 50.5% in Mphuka to 86.0% in Sinyawagora (*χ^2^* = 48.47, p<.001) (Figure 1). Over a third (37%) of households without a child <5 during the distribution campaign had no ITN at the time of the 2008 survey, compared to 19% of households with a child <5 during the campaign (*χ^2^* = 34.20, p<.01) (Table 1). Similarly, a third (34%) of households without a woman who attended ANC within the past 2 years had no ITNs, compared to 20% of households with a woman who attended ANC in the past 2 years (*χ^2^* = 19.44, p<.01). ITN household possession generally increased with household socio-economic status (*χ^2^* = 16.13, p<.01). Euclidian straight-line distance to the nearest rural health center categorized about the mean was not significantly associated with ownership of an ITN at the 5% level; 24% of households 2.5 km or closer to a rural health center had no ITN compared to 29% of households further than 2.5 km from a rural health center (*χ^2^* = 2.88, p = 0.089). Rural health centers with less dense catchment areas generally had lower ITN household coverage levels compared to those with more dense catchment areas (Figure 1).

**Table 1:** Characteristics of households without insecticide-treated mosquito nets, Luangwa District Zambia 2008.

Household characteristic (n = 801)	Percent households (95% Confidence Interval)	Percent households without ITN(95% Confidence Interval)	χ^2^ p-Value
**Child <5 present in household at time of mass distribution**			
Child present	58.9 (55.5–62.3)	18.9 (15.3–22.4)	
No child present	41.1 (37.7–44.5)	37.4 (32.1–42.6)	<0.01
**Presence of a woman in household that visited ANC ≤2 years**			
Yes	51.2 (47.7–54.7)	19.8 (15.9–23.6)	
No	48.8 (45.3–52.2)	33.5 (28.8–38.2)	<0.01
**Household Socio-economic status**			
Most poor	19.9 (17.1–22.6)	32.1 (24.8–39.4)	
More poor	20.5 (17.7–23.3)	28.0 (21.1–35.0)	
Poor	20.0 (17.2–22.7)	27.5 (20.5–34.5)	
Less poor	19.7 (17.0–22.5)	30.4 (23.2–37.6)	
Least poor	20.0 (17.2–22.7)	14.4 (8.9–19.8)	<0.01^§^
**Distance to health facility**			
≤2.5 km	60.5 (57.2–63.9)	24.3 (20.5–28.2)	
>2.5 km	39.5 (36.1–42.8)	29.7 (24.7–34.8)	0.089
**Rural Health Center**			
Chitope	14.2 (11.8–16.7)	24.6 (16.5–32.6)	
High School	7.5 (5.7–9.3)	40.0 (27.2–52.8)	
Kasinsa	18.0 (15.3–20.6)	24.3 (17.2–31.4)	
Katondwe	7.1 (5.3–8.9)	28.1 (16.0–40.1)	
Kavalamanja	3.1 (1.9–4.3)	32.0 (12.3–51.7)	
Luangwa	17.5 (14.8–20.1)	17.1 (10.8–23.5)	
Mandombe	7.7 (5.9–9.6)	22.6 (11.9–33.3)	
Mphuka	12.4 (10.1–14.6)	49.5 (39.5–59.5)	
Sinyawagra	12.5 (10.2–14.8)	14.0 (7.1–20.9)	<001

ITN: insecticide-treated mosquito net.

ANC: Antenatal care clinic.

Logistic regression showed that households without a child who would have been <5 during the campaigns were over twice as likely to not have an ITN during the 2008 survey, as compared to those households with a child <5 during the campaigns [Adjusted odds ratio (AOR)  = 2.43; 95% confidence interval (CI): 1.67–3.55], while controlling for potential confounding factors (Table 2). Households without a woman who attended ANC in the past 2 years were more likely not to own an ITN at the time of the 2008 survey, compared to households with a woman who attended ANC in this interval (AOR  = 1.52; 95% CI: 1.04–2.21). The poorest households were more likely to be without ITNs, compared to those in the least poor wealth quintile (AOR  = 2.75; 95% CI: 1.48–5.12). No significant interaction between distance to health facility and wealth quintile was observed while controlling for the presence of a child <5 and access to ANC (-2LL difference  = 4.91, df  = 4, p>.10). The results were similar when looking at factors associated with households owning any mosquito net.

**Table 2:** Logistic regression analysis* showing factors influencing the lack of insecticide-treated mosquito net household ownership, Luangwa District Zambia 2008.

Household characteristic (n = 801)	Adjusted odds ratio	95% Confidence Interval
**Child <5 present in household at time of mass distribution**		
Child present (reference)	1.00	
No child present**	2.43	1.67–3.55
**Presence of a woman in household that visited ANC ≥2 years**		
Yes (reference)	1.00	
No**	1.52	1.04–2.21
**Household Socio-economic status**		
Most poor**	2.75	1.48–5.12
More poor**	1.97	1.08–3.60
Poor	1.69	0.91–3.14
Less poor**	2.39	1.33–4.30
Least poor (reference)	1.00	
**Distance to health facility (continuous)**	1.01	0.92–1.10

*Rural health center included as a random intercept.

**Significant at the 95% level.

Age ranged from 30 to 80 among the heads of households interviewed in the qualitative portion of the study; 4 heads of household were female, 6 were male. All but 2 of the heads of households were present at the time of the community-wide distribution of ITNs. Three perceived barriers to ITN possession emerged. First, 3 respondents perceived old age as a barrier to obtaining an ITN. One household head reported being away during the free community-wide distribution campaign and asked, “Where do you think an old lady like me will find money to buy one? I don't have any children.” Another woman stated that she could not walk to the distribution point because of the distance, and that the distribution targeted only young children. Second, 2 of 10 household heads indicated that the ITNs they received were now worn out and required replacement. One of the heads of household stated, “I had one [ITN] but it's been long now. It's damaged and not in use.” Third, food security was perceived by 2 of 10 household heads as a more immediate and pressing health concern than malaria. One woman had no desire to talk about ITNs or malaria because she was hungry. She did not accept the free ITN that was offered to her, asking, “What use is this to me? Can I eat it?”

Age of community health workers interviewed ranged from 35–60, and all were male. Interviews with these community health workers confirmed that ITN replacement is a problem. One community health worker pushed for the constant re-distribution of ITNs: “Most of them were happy [the people who received ITNs during the distribution] but a few complained that the ITNs are usually worn out in a short period of time. They needed more; at least after every 3–4 months because the reed mats usually tore the ITNs. If you donors can't manage these 3–4 months you can have distribution every six months.”

Desire for ITNs was not a perceived barrier among the heads of households interviewed. With the exception of the woman suffering from hunger who refused to discuss malaria, heads of households participating in the in-depth interview reported desiring an ITN and accepted a free ITN after the interviews. It is also worth noting that several of the household heads reported that people may falsely state they do not own an ITN in order to increase the likelihood of additional ITNs being distributed in the area.

## Discussion

The Luangwa district has seen great gains in increasing household ITN coverage thanks to the efforts of the Ministry of Health in Zambia, international donors, and local ITN distribution programs. Despite their best efforts to ensure that all households owned at least 1 ITN, approximately 25% of households reported not owning an ITN 2 years after the mass distribution.

Households without children <5 or without a woman who attended ANC in the past 2 years were more likely missed by the ITN distribution campaigns. This result is expected, as households with children <5 had been prioritized to receive ITNs in the past, while routine distribution through ANC targeted pregnant women. Consistent with the quantitative findings, in-depth interviews suggest the elderly are a group that was missed during the mass distribution campaign, as they are without young children and likely not pregnant. Old age was cited as the most commonly perceived barrier to ITN ownership from in-depth interviews with heads of household without ITNs. As such, reliance solely on distribution of ITNs through child health days or to pregnant women through ANC will result in large segments of the population, particularly the elderly without the financial ability to purchase new ITNs, being missed as has been seen in other settings [22].

Consistent with previous research [11], [13], [14], our results showed that wealthier households were more likely to own an ITN, as compared to those in the poorest quintiles. We hypothesize that such households were less likely to be missed by the ITN distribution campaigns in the first place due to increased access to health facilities. Additionally, wealthier households may have been more able to replace worn out ITNs and in general are likely less prone to selling or trading their ITNs for immediate livelihood needs. In order to achieve universal coverage, ITN distribution strategies will need to ensure that the poorest households and households with limited access to distribution points are not missed in distributions. Otherwise pockets of the population will remain unprotected. One option for addressing this issue would be to have community health workers identify and provide ITNs to households within their catchment areas that are missed by mass-distribution and routine ANC campaigns.

While approximately 16,000 ITNs were reported delivered in 2005–2006, we estimate from the number of reported or observed ITNs in the households at the time of 2008 survey that only 6,000 were in circulation at that time. It remains unknown how many ITNs were delivered and wore out before the surveys were taken, however the in-depth interviews with community health workers suggest that ITN wear and tear occurs at a high rate in this setting. Other possible explanations for a discrepancy of this magnitude include: ITNs may have been delivered to the district but not to households; ITNs may have been delivered to households and then were traded or sold out of the district; or households may be presenting false information regarding their ownership of an ITN. Personal communication with program officers revealed that some ITNs are waiting in storage units for distribution, but most likely a combination of these 4 reasons is occurring. The rate of ITN replacement is one factor which national malaria control programs and international donors can influence greatly, but it is also an area that has garnered little research as to which ‘keep-up’ strategy will be most effective. It is generally understood that LLINs remain efficacious for at least 3 years, however more understanding regarding how long these LLINs remain viable in actual living conditions is needed to inform ITN procurement and distribution systems and maintain high population coverage. Different settings have seen a large number of ITNs deteriorate before the 3-year period [23], [24]. Another way to influence the rate at which ITNs deteriorate is by altering their design. Like many rural populations in Africa, the people of Luangwa district typically sleep on reed mats with sharp edges that can easily tear the fine mesh material of ITNs. ITN manufacturing must do more to bolster the net's bottom border – more durable fabric along this bottom hem would likely increase the lifespan of ITNs.

There are several limitations to this study worth noting. Information bias, as a result of misreporting the number or presence of ITNs and/or the date of retreatment or purchase of ITNs, may have affected our coverage estimates. As supported by our qualitative results, households may have falsified their ITN ownership status in hopes of getting additional nets. However, misclassification of ITNs as untreated nets was likely minimal as the analysis for any type of mosquito net produced similar results. The individual in-depth interviews discussed here were from a small sample. Perceived barriers to ITN ownership from other households not interviewed may differ from what is reported here, as these results are not intended to be a comprehensive list of reasons why a household may not have an ITN. The individual in-depth interviews did not ask questions regarding the selling or trading of ITNs, and so the reasons for the discrepancy between ITNs delivered and ITNs available remain speculative. Euclidian distance to the nearest rural health facility may not be the best proxy of access to information regarding ITN distribution and access to distribution points themselves. Luangwa district has a series of community health worker posts that may have disseminated information regarding ITNs, as well as acted as distribution points. Household education levels were dropped from the analysis because education as measured by the household survey was homogeneous across households (i.e. all heads of households had received some primary education). Mother's education could also potentially influence whether or not a household had an ITN, however our analysis included households without children or mothers, making mother's education unsuitable.

The greatest reduction in the malaria burden will be seen as household ITN ownership approaches 100%, with a focus of ITN use on all children, pregnant women and adults [25]. However, ITN distribution does not necessarily translate into coverage, as has been seen in Luangwa where enough ITNs were distributed to achieve 3 ITNs per household, but household ITN coverage was only 73.5%. In order to go beyond the coverage achievable through convenience distribution programs (e.g. at ANC clinics and child-health days), ITN distribution strategies should seek to specifically target households who have been missed as a result of the distribution design. Maximum ITN coverage can be achieved if specific attention is paid to individual households in the area that do not otherwise qualify for an ITN through existing mechanisms. Cooperation with local institutions such as community health workers, village headmen, churches, and schools could help identify those households that are typically missed by routine ITN distribution and redistribution campaigns.
